# The Ohio State Dental Society

**Published:** 1871-12

**Authors:** 


### Editorial.

### THE OHIO STATE DENTAL SOCIETY.

The sixth annual meeting of this Society has just been held. There was a large attendance of both members and visitors. The sessions continued during three days and two evenings, and throughout the whole a very great interest was maintained. The discussion of the various topics presented gave evidence of progressive thought and research in every direction from which tribute may be derived for the advancement of our profession. There was a disposition to change somewhat from the almost universal routine of proceeding—so far, at least, that certain foundation principles of the profession, as such, were taken up and very fully discussed; and among these was the subject of the elevation of the profession in our State. As a result of this, the Society selected and appointed three persons, who were presumed to be well qualified for the work, to serve the profession in the State for the ensuing year as Normal-class or Clinical teachers, whose duty it will be to go wherever called, by a sufficient number of the profession in any locality, and give clinical demonstrations in dental practice. From such a work, if fully carried out and well conducted, great good will result. The profession in several places are already making arrangements to avail themselves of the advantages afforded by this arrangement.

To give variety and add a new feature to the proceedings, a general quiz of one hour was held twice each day, thus drawing out from each and every member valuable thoughts, ideas and suggestions upon points of practice and principles, and in this way making every one take part, and contribute something. There were but few papers read, but this deficiency was not marked, from the fact that a great interest was maintained throughout the meeting. The papers read, however, were excellent.

There are two directions in which this Society is putting forth a strong effort. The one is professional education, and the other regulation and protection—these both having reference to those now in the ranks, as well as to those who enter hereafter. These are subjects that all State societies should consider and act upon. The Board of Examiners acting for and under the control of this Society, and under the authority of law, is accomplishing much for the profession and the public in this State.

Taking all things into consideration, we regard this as the best and most efficient meeting of the Ohio State Dental Society ever held. T.

